# Real-Time PCR Assay to Quantify Moloney Murine Leukemia Virus in Mouse Cells

**DOI:** 10.3390/microorganisms13061268

**Published:** 2025-05-29

**Authors:** Jiwon Choi, Amaiya Murphy, Takayuki Nitta

**Affiliations:** 1 Department of Biology, Savannah State University, Savannah, GA 31404, USA; jchoi1@student.savannahstate.edu (J.C.); amurph16@student.savannahstate.edu (A.M.); 2 Department of Molecular Biology and Biochemistry, Cancer Research Institute, University of California, Irvine, CA 92697, USA

**Keywords:** murine leukemia virus, quantitative PCR, focal immunofluorescence assay, exogenous retrovirus, endogenous retrovirus, viral DNA

## Abstract

Murine leukemia viruses (MuLVs) are retroviruses that cause various diseases in mice and have served as a model for retrovirus replication and pathogenesis. MuLVs are separated into infectious exogenous retroviruses (XRVs) and endogenous retroviruses (ERVs) that are remnants of ancient infectious XRVs. Detection of XRVs in the original host cells has some difficulties because of the high similarity in sequence between ERVs and XRVs and expression of some ERV genes. There are some techniques available for monitoring retroviral replication, but each comes with limitations in terms of labor intensity, detection range, cost, and phases after infection. This study developed a novel quantitative PCR (qPCR) method for assessing replication of Moloney MuLV (M-MuLV) in mouse cells. The method amplified the region in packaging signal and *gag* and distinguished exogenous M-MuLV from ERVs with mouse SC-1 cells. The qPCR system could quantify viral sequences in infected cells from 16 to 72 h post-infection, with a 3-log range of difference. This was compared with the traditional infectivity assay, focal immunofluorescence assay. In conclusion, the developed qPCR system provides a rapid, sensitive, and scalable alternative for quantifying M-MuLV infectivity, with potential for broader applications in MuLV research.

## 1. Introduction

Murine leukemia viruses (MuLVs) are gamma retroviruses that cause various diseases in mice, including lymphoid and hematopoietic tumors, erythroproliferative disease, and neurological disorders [1,2]. Moloney murine leukemia virus (M-MuLV) is a well-studied replication-competent oncogenic retrovirus, and the virus has enabled the understanding of general phenomenon of leukemogenesis and served as a model for studying retrovirus replication and pathogenesis [1,2]. MuLVs carry two copies of positive-sense single-stranded RNA that encode three essential genes: *gag*, which encodes the structural proteins; *pol*, which produces the critical enzymes required for retroviral replication, including protease, RT, and integrase; and *env*, which encodes the surface and transmembrane proteins in viral envelope that work together to mediate the entry of an infectious virus particle into a new host cell [2]. The replication cycle begins with the binding of the viral Env protein to host cell receptors, leading to membrane fusion. Following entry, the viral enzyme reverse transcriptase converts the RNA genome into linear double-stranded DNA within the cytoplasm [3]. The resulting viral DNA integrates into the host genome as a provirus. The viral RNA is transcribed from the provirus and the translated proteins assemble to produce progeny virions.

MuLVs are identified in mice as infectious exogenous viruses (XRVs) and as endogenous retroviruses (ERVs). ERVs are the remnants of ancient XRVs that once infected the genomes of ancestral organisms. These retroviruses integrated into the host’s germline DNA and became stable parts of the genome. While most of these viral sequences have been inactivated by mutations or deletions, some ERVs still retain functional sequences and influence biological activities in hosts [4,5]. MuLVs have three host range subgroups, ecotropic MuLVs (E-MuLVs), xenotropic MuLVs (X-MuLV) and polytropic MuLVs (P-MuLV), and they are determined by variation of their sequence and receptor usage [6]. Ecotropic XRVs (E-XRVs) were discovered as causative agents of leukemias or lymphomas in the common inbred mouse strains and their tropism is limited to the mice due to expression of their receptor, mouse cationic amino acid transporter 1 (mCAT1) [7,8,9]. Both xenotropic and polytropic XRVs (X-XRVs and P-XRVs) use the XPR1 receptor expressed in mouse and other mammalian cells. Laboratory mice carry multiple copies of ERVs resulting from germline integrations of xenotropic MLVs (Xmv loci), polytropic MLVs (Pmv and Mpmv (modified polytropic) loci) and in some strains of ecotropic MLVs (Emv loci) [6].

Techniques for measuring proteins, nucleic acids, and enzymatic activities in viruses are crucial for gaining insights into viral replication. Several methods are available for detecting and quantifying XRVs, including focal immunofluorescence assay (FIA), enzyme-linked immunosorbent assay (ELISA), reverse transcription polymerase chain reaction (RT-PCR), and reverse transcriptase (RT) assays. FIA is a time-consuming, immunofluorescence-based technique that visualizes foci on dishes inoculated with viruses, allowing for the assessment of the number of infectious virus particles in samples [10]. ELISA is a less time-consuming technique that can detect viral antigens, but this technique requires a specific antibody and is often expensive and limited by a narrow linear range of antigen concentration [11]. RT-PCR offers greater sensitivity and the ability to detect low concentrations of viral RNA. RT assay directly measures the activity of reverse transcriptase, an enzyme uniquely found in the retroviridae family by quantifying its ability to synthesize cDNA from RNA templates. In addition to the traditional RT assay, product-enhanced reverse transcriptase (PERT) assays that integrated the real-time PCR was also developed to amplify cDNA produced by retroviral RT [12]. These methods have been applied to detect types of retroviruses in biological products and to monitor replication of retroviruses in cells and animals.

The detection of XRVs in mouse cells can be challenging because of the presence of ERVs. Due to the high sequence similarity between ERVs and XRVs, expression of a portion of ERVs, and antibody cross-reactivity, both nucleic acid-based and antibody-based detection need high specificity to distinguish subtle differences between the two. Since M-MuLV is used widely as a model retrovirus in molecular biology and virology research, we aimed in this study to establish a PCR-based virus detection system using M-MuLV and three mouse cell lines of distinct origins. We also compared the established PCR-based method to the traditional FIA. Our approach utilizing a primer pair targeting the packaging signal (psi) and *gag* region offers a rapid, sensitive, and quantitative alternative to conventional FIA assay, enhancing the detection and monitoring of M-MuLV replication in mouse cells.

## 2. Materials and Methods

### 2.1. Cells

The mouse fibroblast cell lines, SC-1, BALB/3T3, NIH/3T3, and 43D cells were cultured with DMEM media containing 10% fetal bovine serum, 100 IU/mL of penicillin and 100 μg/mL of streptomycin (Corning Incorporated, Corning, NY, USA) at 37 °C with 5% CO_2_. SC-1 cells are derived from a mouse in California and show high sensitivity to both N- and B-tropic MuLVs [13]. BALB/3T3 were derived from a single pool of 14- to 17-day-old Balb/c mouse embryos [14]. NIH/3T3 cells are derived from NIH Swiss mouse embryo culture [15]. The mouse cell lines, SC-1 (CRL-1404) and BALB/3T3 (CCL-163) were obtained from the American Type Culture Collection (Manassas, VA, USA). Establishment of 43D mouse fibroblast cell line stably infected with the wild-type M-MuLV was described previously [16].

### 2.2. Preparation of 43D Viruses

The preparation of 43D viruses is described previously [16]. In this study, 7 × 10^5^ 43D cells were seeded on 10 cm dishes and incubated for 48 to 60 h. The collected viruses were filtered using 0.45 μm syringe filters, pooled, and concentrated by ultracentrifugation with a 20% sucrose cushion. The pelleted viruses were resuspended in phosphate-buffered saline (PBS), filtered again through 0.45 μm syringe filters and, aliquots of the concentrated viruses were frozen at −80 °C.

### 2.3. Plasmid, DNA Extraction and Polymerase Chain Reaction

A molecular clone p63-2 was established by cloning of M-MuLV provirus in the M-MuLV clone A9 cells [17]. The plasmid is 14.1 kbps in length [18] and it was digested with EcoRI (New England Biolabs, Ipswich, MA, USA). Mouse DNA was extracted from SC-1, BALB/3T3, NIH/3T3 and 43D cells. The cells were washed three times with phosphate-buffered saline (PBS) and lysed and incubated with the lysis buffer containing 100 mM of NaCl, 10 mM of Tris-HCl (pH 8.0), 25 mM of EDTA, 0.5% SDS 20 μg/mL of DNase-free RNase A and 100 μg/mL of proteinase K at 65° C for overnight. The genomic DNA was extracted with phenol, phenol-chloroform and ethanol precipitation.

The PCR reaction mixtures had 20 μL with 1× GoTaq Master Mixes (Promega, Madison, WI, USA), 0.5 μM of each forward and reverse primer (Table 1) and 50 ng of the mouse DNA samples or 1 ng of p63-2 DNA. The PCR and quantitative PCR (qPCR) conditions were as follows: 95 °C for 5 min, 40 cycles of 95 °C for 30 s, 55 °C for 30 s and 72 °C for 30 s, followed by a final extension of 72 °C for 5 min. The PCR products were resolved by agarose gel electrophoresis.

The qPCR reaction mixtures had 20 μL with 1× GoTaq Master Mixes, 0.5 μM of each forward and reverse primer (Table 1), 1× Evagreen dye (Biotium, Inc., Fremont, CA, USA) and 300 ng of the mouse DNA samples for *gag* detection or 50 ng of the mouse DNA samples for detection of mouse *c-myc*. Each reaction was performed in triplicate. Data amplification was analyzed by the qPCR quantification software (Azure Cielo Real-Time PCR, Azure Cielo Manager Ver 1.0.8.26). The average weight of a single DNA bp was regarded as 650 Daltons. The number of DNA copies in 1 ng of pHA-gg188 (6 kbps) was calculated as 1.52 × 10^8^. The standard DNA was prepared with the pHA-gg188 digested with EcoRI containing 1 ng/μL of salmon sperm DNA. The copy number of c-*myc* in 1 ng of mouse genomic DNA was calculated as 333.

### 2.4. Focal Immunofluorescence Assay (FIA)

Frequency of viral infection was measured by the FIA described previously [19] with slight modification. Briefly, 2 × 10^5^ SC-1 cells were seeded per 6-cm plate 4 h prior to infection. Serial dilutions of the 43D viruses were then adsorbed to the cells in the presence of 8 μg/mL of hexadimethrine bromide (MilliporeSigma, St. Louis, MO, USA) for 2 h, followed by the addition of growth medium. At 16 to 72 h post-infection, cells were washed three times with PBS and then fixed with 4% paraformaldehyde (MilliporeSigma). The fixed cells were washed three times with PBS, permeabilized with 1% Triton-X100 and incubated with the blocking buffer containing 10% calf serum in PBS for 2 h at room temperature. The serum against MuLV p30^CA^ [20] and a goat anti-rabbit IgG conjugated with Alexa Fluor 488 (Thermo Fisher Scientific, Waltham, MA, USA) were used for visualizing the foci, clusters of the infected cells. The nuclei were visualized by counterstaining with 1 μg/mL Hoechst 34580 (BD Biosciences, East Rutherford, NJ, USA) for 15 min. The images were obtained and analyzed by an LSM800 microscope system (Carl Zeiss, San Diego, CA, USA) and a fluorescent microscope, IN480TA-FL-MF (Microscope Central, Feasterville, PA, USA). The clusters of cells having 8 or more infected cells were regarded as foci.

### 2.5. Reverse Transcriptase Assay

Reverse transcriptase activity was measured with EnzChek™ Reverse Transcriptase Assay Kit (Invitrogen, Waltham, MA, USA). To determine the RT activity in the viruses prepared from 43D cells, a standard curve was established with the M-MuLV reverse transcriptase (APExBIO, Houston, TX, USA). The poly(A) ribonucleotide template (5 μL) and oligo d(T)16 primer (5 μL) were mixed in a nuclease-free microfuge tube and incubated at room temperature for 1 h to allow annealing. The reaction mixture (template/primer) solution was then diluted into 2.0 mL of polymerization buffer. For each sample, 20 µL of the reaction mixture was aliquoted and diluted with enzyme dilution buffer containing 50 mM Tris-HCl (pH 7.6), 20% glycerol, and 2 mM dithiothreitol. A standard curve was prepared using known concentrations of RT, ranging from 20 U/µL to 0.125 U/µL, through serial dilution in enzyme dilution buffer. 5 μL of the diluted enzyme standards and samples were added to the wells of a 96-well plate containing the reaction mixture, with a control well of enzyme dilution buffer (5 μL). The reaction samples were incubated at 37 °C for 1 h, and the reaction was stopped by adding 2 μL of 200 mM EDTA to each well. For fluorescence measurement, a 1× TE buffer was prepared by diluting 20× TE buffer with nuclease-free water. The working solution was prepared by adding 50 μL of PicoGreen reagent to 17.2 mL of 1× TE buffer. A total of 173 µL of the PicoGreen working solution was added to each reaction well, followed by a 5-min incubation at room temperature. The fluorescence of the samples was measured using a microplate reader (SLXFA-SN, Agilent Technologies, Santa Clara, CA, USA) with an excitation wavelength of 480 nm and an emission wavelength of 520 nm. Data were analyzed by BioTek Gen5 Software (Agilent Technologies). The standard curve for M-MuLV RT was established using Microsoft Excel.

### 2.6. Statistical Analysis

The efficiency and accuracy of the standard curves used to quantify the copy number of *gag*/psi and mouse c-*myc* were evaluated by analyzing the coefficient of determination (R²). Similarly, the correlation between the amount of inoculated virus and the resulting relative copy number of *gag*/mouse c-*myc* in SC-1 cells was assessed using R².

To evaluate the qPCR system for measuring viral replication after multiple rounds of infection, the ratio of *gag* to c-*myc* at 72 h post-infection relative to 16 h post-infection was compared across cells exposed to different viral doses using Student’s *t*-test.

## 3. Results

**Figure 1:**
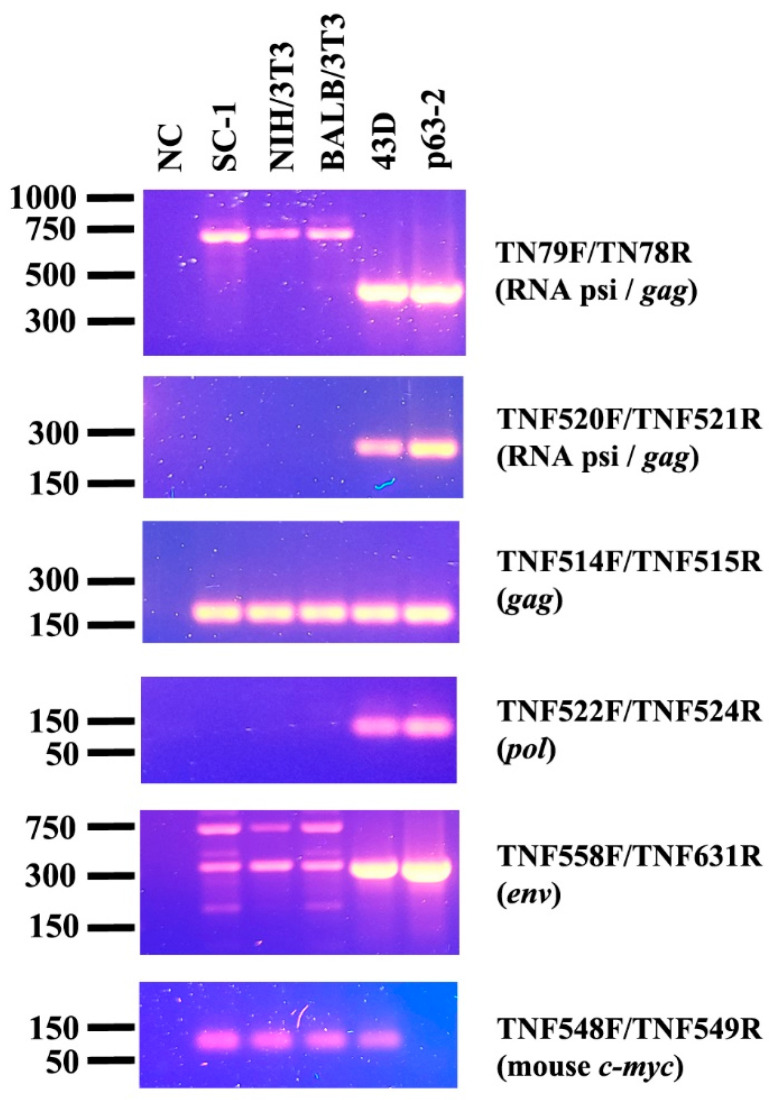
Amplification of MuLV sequence by PCR. MuLV genes were amplified by sets of primers located in the psi, *gag*, *pol* and *env* regions. The uninfected mouse cell lines (SC-1, NIH/3T3, BALB/3T3), the MuLV-infected 43D cells and the plasmid, p63-2 encoding the entire M-MuLV were subjected to PCR. Mouse c-*myc* was amplified to assess the quality of the DNA samples.

To establish a new PCR-based method for detecting M-MuLV DNA in mouse cells, five primer sets were designed in the regions of psi, *gag*, *pol* and *env* (Table 1). DNA was extracted from the 3 uninfected mouse cell lines with the different origins, SC-1, BALB/3T3 and NIH/3T3. The DNA extracted from 43D cells, NIH/3T3 cells stably infected with M-MuLV, and a molecular clone encoding M-MuLV were used as positive controls. The primer sets TNF520F/521R showed specific amplification of M-MuLV DNA from 43D cells and p63-2 and showed no amplification from the uninfected mouse cell lines (Figure 1). The primer set TNF522F/524R showed a minor amplification from NIH/3T3, BALB/3T3 and SC-1 cells. Amplification of M-MuLV DNA from all the samples was comparable with the primer set TNF514F/515R. The longer PCR products were amplified in the DNA samples isolated from the uninfected mouse cells than those from 43D cells using the primer sets, TN79F/TN78R and TNF558F/TNF631R. Mouse c-*myc* was used for checking quality and amount of the DNA samples isolated from the cells. These results indicated that TNF520F/521R was the most reliable primer set for specifically detecting exogenous M-MuLV DNA in mouse cells.

**Figure 2:**
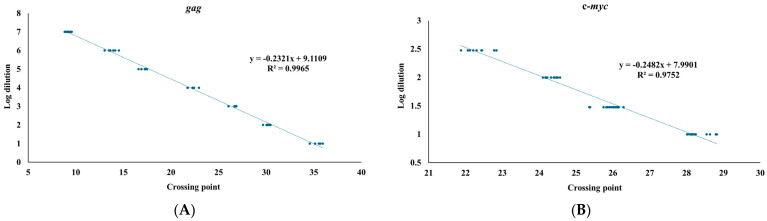
Standard curves for *gag* and c-*myc*. (**A**) MuLV psi and *gag* region was amplified with pHA-gg188. Crossing point (x-axis) and a series of 10-times serial dilution ranging from 1 ng to 1 fg are shown in the log dilution (y-axis). (**B**) An internal control, mouse c-*myc*, was amplified with the DNA samples extracted from SC-1 cells. Crossing point (x-axis) and a series of serial dilutions ranging from 10 ng to 300 ng (y-axis) are shown in the log dilution. Standard curves for *gag* and mouse c-*myc* were generated from five and nine independent qPCR runs, respectively, each performed with triplicate technical replicates. R² values represent the coefficients of determination for the standard curves.

We next employed to establish a qPCR system with the primer set TNF520F/521R and Evergreen dye. For assessing sensitivity of the qPCR system, pHA-gg188 encoding a portion of psi and *gag* was prepared with a serial dilution technique (Figure 2). The doses of the plasmid samples well correlated with the crossing points automatically made by the qPCR quantification software, demonstrating that the qPCR system could quantify copy number of *gag* genes in the sample ranging from 1 fg to 1 ng of pHA-gg188. To account for variations in DNA input and to ensure accurate measurement of viral replication in the infected cells, a serial dilution ranging from 10 ng to 300 ng DNA extracted from the SC-1 cells were used to establish the standard curve for mouse c-*myc* copies (Figure 2). The qPCR system showed correlation between the doses of DNA and the crossing points.

**Figure 3:**
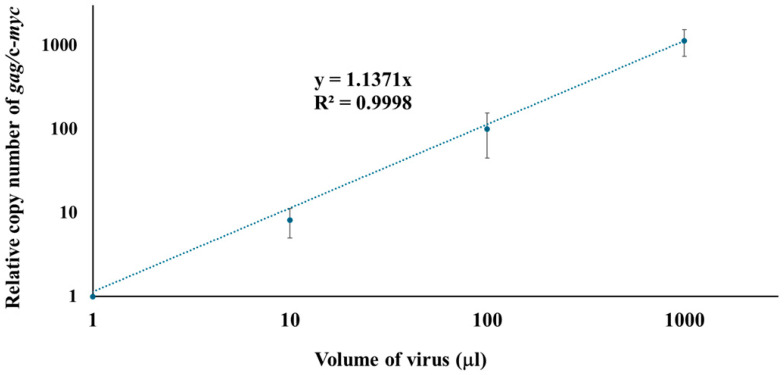
Correlation between inoculated virus volume and copy number of *gag/*c-*myc* in SC-1 cells. Doses ranging from 1 µL to 1000 µL of viruses were inoculated into uninfected SC-1 cells. DNA was isolated from the cells 16 h post-infection. The relative copy number of *gag*/mouse c-*myc* was determined by qPCR. Each condition was tested in four or more biologically independent infection experiments (*n* = 8 for 1 µL, 10 µL, and 100 µL; *n* = 4 for 1000 µL). For each biological replicate, qPCR was performed in triplicate. Ct values from technical replicates were averaged to generate a single value per biological sample. The final average and standard deviation were calculated across 4–8 biological replicates. The averages and standard deviations (bars) are shown. R² values represent the coefficients of determination for the standard curves.

To investigate the early phase of viral infection, the qPCR system developed in this study was used to examine the relationship between viral dose and the copy number of viral DNA. For this purpose, viral doses ranging from 1 µL to 1000 µL were inoculated into uninfected SC-1 cells, and DNA was isolated from the cells 16 h post-infection. The copy number of the *gag* was normalized to the copy number of mouse c-*myc* to quantify viral replication. As shown in Figure 3, the amount of viral DNA was strongly correlated with the volume of inoculated 43D virus within the tested range. To estimate viral input volumes for unknown samples in future experiments, we characterized the M-MuLV viruses used in this study by measuring reverse transcriptase activity using the EnzChek™ RT assay kit with a commercial MuLV reverse transcriptase as a reference. The RT activity of the 43D viruses used in this study was 1.024 units per 1 mL.

**Figure 4:**
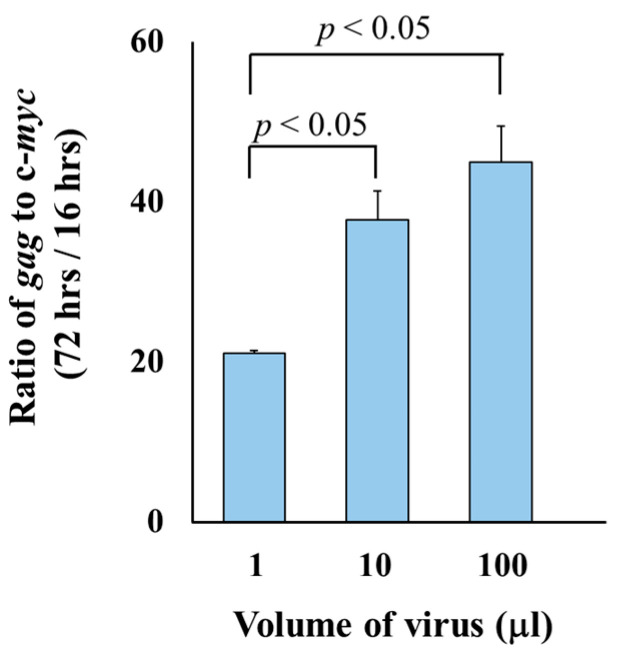
Comparison of *gag*/c-*myc* ratios at 16-h and 72-h post-infection. Doses ranging from 1 µL to 100 µL of viruses were inoculated into uninfected SC-1 cells. DNA was isolated from the cells at 16 h and 72 h post-exposure. The viral replication was determined by calculating the ratio of *gag* to c-*myc* copy numbers at 72 h post-infection relative to 16 h post-infection. Each condition was tested in two biologically independent infection experiments. For each biological replicate, qPCR was performed in triplicate. Ct values from technical replicates were averaged to generate a single value per biological sample. The final average and standard deviation (SD) were calculated across the two biological replicates. The ratios of *gag* to c-*myc* copy numbers were compared between time points using Student’s *t*-test.

To evaluate the potential applications of the qPCR system for monitoring multiple rounds of infection, we compared viral replication in the samples collected at 16- and 72-h post-infection. The copy numbers of *gag*/*c-myc* varied between the two time points depending on the viral dose, showing approximately 21-, 38-, and 45-fold increases when infected with 1, 10, and 100 µL of virus, respectively (Figure 4). The ratio of *gag* to c-*myc* copy numbers at 72 h versus 16 h post-infection differed significantly in the cells exposed to 1 µL compared to 10 µL, as well as between 1 µL and 100 µL of viruses. No statistically significant difference was observed between the ratios in the cells exposed to 10 µL and 100 µL of the viruses.

**Figure 5:**
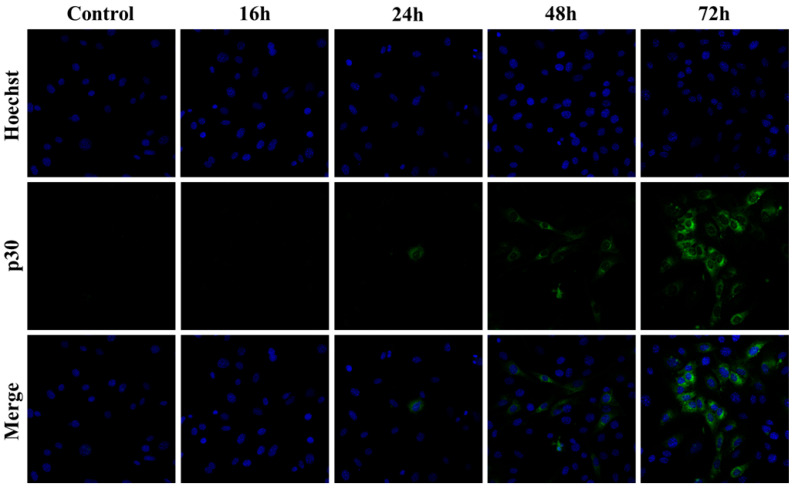
Detection of viral infection foci in SC-1 cells by FIA. SC-1 cells were inoculated with the virus isolated from 43D cells and incubated for 16 to 72 h. The cells were fixed and intracellular Gag proteins were visualized with anti-p30^CA^ and anti-rabbit IgG conjugated with Alexa Fluor 488. The nucleus was counterstained with Hoechst. Representative images from independent fields of view were captured from separate samples fixed at 16, 24, 48, and 72 h post-infection. Control indicates the cells without infection.

The focal immunofluorescence assay (FIA) is a traditional method to measure infectivity by visualizing the clusters of infected cells with the primary antibodies against viruses and the secondary antibodies conjugated with fluorochrome [16]. This method depends on the spread of virus infection rather than a visible cytopathic effect and can be applied for any adhesion cells that are susceptible to viral infection. To compare sensitivity of FIA and the qPCR at different timepoints after infection, SC-1 cells were inoculated with the viruses isolated from 43D cells and incubated for 16 to 72 h. Capsid (a part of poly-Gag) protein in the infected cells was stained to visualize the foci. At early time points (16–24 h post-infection), no visible foci were observed, suggesting that viral replication and spread were below detection levels at stage (Figure 5). However, by 72 h post-infection, clear foci were present against a negative background of uninfected cells, confirming active viral replication and cell-to-cell transmission. Inoculation of 0.1 µL to 10 µL of the 43D virus yielded 10 to 1000 foci, and the average of infection events (foci/1 mL) was 1.07 × 10^5^ (Table 2). The foci aggregated on the dishes with 100 µL of the 43D virus, making them uncountable.

Infection events/cell were measured by FIA and qPCR. The same virus stock was used for comparing the viral replication in the assays. In the FIA, the clusters of cells having 8 or more infected cells were regarded as foci. The two or more infection experiments were conducted with doses of the viruses. The averages and standard deviations of foci/6 cm dish are shown. The infection events/cell measured by FIA assay (average) were calculated as the number of foci per the number of target (2 × 10^5^) cells. The infection events/cell measured by qPCR (average) were calculated as the copy number of *gag*/2 × c-*myc*.

## 4. Discussion

In this study, we developed a novel qPCR system to quantify the replication of M-MuLV and compared it with the FIA technique, which is commonly used to assess retrovirus replication [16]. The qPCR system utilizing the unique primer pair distinguished M-MuLV from ERVs in mouse SC-1, BALB/3T3, NIH/3T3 cells [21,22,23]. This system allows the quantification of the *gag* sequence in infected cells as early as 16 h post-infection with a 3-log difference, in contrast to the traditional FIA, which requires several days to measure MuLV infection.

ERVs comprise a substantial portion of vertebrate genomes, including those of humans, cats, sheep, and mice [5]. Over evolutionary time, most ERVs accumulate mutations that render them replication-incompetent. However, many ERVs still retain the capacity to be transcribed and can influence infection of ERVs through mechanisms such as recombination, tolerance induction, receptor interference and antiretroviral restriction factors [5,6]. The sequence similarity between ERVs and XRVs can also interfere with the detection and characterization of ERV genomes [24]. In this study, the amplification of the M-MuLV sequence was assessed using five primer sets targeting different genomic regions across three mouse cell lines of distinct origins (Figure 1). Among the primer sets tested in the study, the region spanning psi and *gag*, especially for the sequence encoding glyco-*gag* unique peptide (NCBI Refseq J02255, nt 550–802) [25] showed specific amplification by PCR. Our comparative sequence analysis using the region showed that exogenous MuLVs share high similarity with M-MuLV. In contrast, M-MuLV exhibited low similarity to endogenous MuLVs, particularly at the 5′ and 3′ regions (Supplementary Figure S1 and Table S1). Our findings are also consistent with a previous study that demonstrated psi-*gag* region is highly conserved among exogenous MuLVs but divergent in endogenous elements [19].

Friend virus susceptibility 1 (Fv1) is an ancient retroviral Gag derivative that confers differential restriction activity depending on variations in the viral capsid [21]. Different alleles of *Fv1* mediate tropism-specific restriction: Fv1ⁿ restricts B-tropic MuLV, Fv1ᵇ restricts N-tropic MuLV, while Fv1^null^ does not restrict either variant. Since the primer pair TNF520F and TNF521R did not produce nonspecific amplification in NIH/3T3 and BALB/3T3 cells, which harbor the *Fv1ⁿ* and *Fv1ᵇ* alleles, respectively (Figure 1), our qPCR assay can quantify viral replication while reflecting Fv1-mediated restriction. In the current study, SC-1 cells were used to characterize the qPCR assay and to compare it with the FIA assay because the SC-1-based qPCR system is expected to have broader applicability for measuring infection event and replication of various MuLV tropisms, owing to its high sensitivity to both N- and B-tropic MuLVs [13].

While FIA is a reliable tool for assessing viral replication based on viral spread in cell culture, one limitation is the time required after infection [16]. As shown in Figure 5, short exposure to the virus did not produce visible foci, and reliable signals in the system typically emerged 72 h post-infection. The FIA also required a relatively long time to count foci under the microscope and specific antibodies targeting viral antigens. The qPCR system established in the study could detect viruses in infected cells at 16 h post-infection, and the incubation time would be further shortened by optimizing experimental conditions, such as cell number and virus dosage. Quantification by the qPCR also does not require special techniques, and a large number of samples can be processed quickly using commercially available basic materials.

Although the qPCR system has an advantage to detect viruses in the cells with short exposure, we also found that the system can be applied for long term incubation (Figure 4) due to its ability to detect a wider range of differences. The relative extent of viral infection, determined by comparing the *gag* to c-*myc* copy number ratios between samples collected at 72 and 16 h post-infection, was influenced by the viral dose. Inoculation with 1, 10, and 100 µL of virus resulted in approximately 21-, 38-, and 45-fold increases in relative extent of viral infection, respectively (Table 2). The data suggested that the qPCR system could measure the effect of exposure time on viral replication for a single dose, but it requires optimization for comparing samples with different exposure times and doses.

The current study also provided insights into the sensitivity ranges of the two methods (Table 2). In the FIA system, visible and individual foci can be observed with virus volumes ranging from 0.1 to 10 µL. In contrast, the copy number of *gag*/c-*myc* was quantifiable with virus volumes ranging from 1 to 1000 µL by qPCR. The data demonstrated that our qPCR system covered a wider range of differences but was less sensitive in assessing extent of viral infection compared to the FIA. The limitation of sensitivity in qPCR system with low viral dose is likely due to the sensitivity threshold of *gag* detection, as the assay’s minimum detectable quantity is 1 fg of HA-gg88, corresponding to approximately 150 copies of *gag* (Figure 2). Additionally, low viral doses might result in reduced efficiency of viral binding to target cells and/or reverse transcription. The limitation of sensitivity could be addressed by implementing nested PCR or increasing the amount of template DNA used in the assay. Our comparative sequence analysis (Supplementary Figure S1 and Table S1) identified a conserved region within the psi/*gag* sequence among exogenous MuLVs. Insights from this alignment could inform future optimization of the qPCR assay and enhance its sensitivity in subsequent studies.

Since both methods could measure viral signals in the cells with 1 and 10 µL of virus, infection events/cell measured by the FIA (number of foci per target cell) were compared to those measured by qPCR (*gag*/2 × c-*myc*). The results were comparable between the doses, supporting the reliability of the qPCR data. The data also implied that approximately 80 copies of *gag* gene could be required at 16 h post-infection to establish a visible focus at 72 h post infection.

## 5. Conclusions

The qPCR-based detection system developed in our study effectively distinguishes M-MuLV from background ERVs by utilizing the unique primer set and SC-1 cells. The qPCR system enabled the quantification of viral sequences in infected cells from 16 to 72 h post-infection with a range of a 3-log difference. This method demonstrated a broader range of detection differences and a wider post-infection duration compared to the traditional FIA method. In addition, our qPCR system could offer the advantage of processing a large number of samples using commercially available materials. The information about RT activity in the virus will also support determination of the volume of unknown samples to be used in the qPCR system. In conclusion, the qPCR method developed in the study could be a valuable tool for quantifying M-MuLV infectivity in mouse cells.

## Figures and Tables

**Table 1:** Summary of the primers used in this study.

Target/ NCBI Refseq	Primer Name	Location/Gene	Sequence
MuLV/	TN79F	nt 274–291/RNA psi	5′—gcgtcggtactagttagc
J02255	TN78R	nt 700–683/*gag*	5′—gactggttgtgagcgatc
	TNF520F	nt 550–569/RNA psi	5′—cgcgtcttgtctgctgcagc
	TNF521R	nt 802–785/*gag*	5′—atgaggtctcggttaaag
	TNF514F	nt 2043–2062/*gag*	5′—agcaagctattggccactgt
	TNF515R	nt 2230–2211/*gag*	5′—tctagggtcaggagggaggt
	TNF522F	nt 4591–4610/*pol*	5′—cagacacctctaccctcctc
	TNF524R	nt 4721–4702/*pol*	5′—gacccaatacttctttgttt
	TNF558F	nt 5771–5794/*env*	5′—actgacatggcgcgttcaacgctc
	TNF631R	nt 6279–6296/*env*	5′—aggcacagtagaaggagt
mouse c-*myc*/	TNF548F	nt 1731–1750	5′—gagctgaagcgcagcttttt
NM_001177352	TNF549R	nt 1796–1777	5′—ggccttttcgttgttttcca

NCBI accession numbers of genes, primer names, locations of nucleotide sequence in each gene and sequence of nucleotide are shown.

**Table 2:** Comparison of viral replication measured by FIA and qPCR.

Inoculated Virus (Volume)	0.1 μL	1 μL	10 μL	100 μL
Number of foci/6 cm dish	12.5 + 2.5	114.0 + 8.3	833.5 + 8.5	TMTC *
Infection events/cell measured by FIA (Number of foci/Number of target cell)	6.3 × 10^−5^	5.7 × 10^−4^	4.2 × 10^−3^	NA *
Infection events/cell measured by qPCR (*gag*/2 × *c-myc*)	NA *	4.2 × 10^−2^	3.4 × 10^−1^	4.5 × 10^0^
Ratio of infection events/cell (qPCR/FIA)	NA *	74.0	81.1	NA *

* TMTC, too many foci to count, NA, not available due to either lack of stable signals (qPCR) or too many foci to count (FIA).

## Data Availability

The data presented in this study are available from the corresponding author upon request.

## Supplementary Materials

The following supporting information can be downloaded at: https://www.mdpi.com/article/10.3390/microorganisms13061268/s1, Figure S1: Graphic summary of comparative sequence analysis between M-MuLV and other MuLVs [26]; Table S1: Summary of comparative nucleotide sequence analysis among MuLVs.

## Abbreviations

The following abbreviations are used in this manuscript:

MuLVs	Murine Leukemia Virus
M-MuLV	Moloney Murine Leukemia Virus
E-MuLV	Ecotropic Murine Leukemia Virus
X-MuLV	Xenotropic Murine Leukemia Virus
P-MuLV	Polytropic Murine Leukemia Virus
mPmv	Modified Polytropic
XRVs	Exogenous Retrovirus
ERVs	Endogenous Retrovirus
FIA	Focal Immunofluorescence Assay
ELISA	Enzyme-Linked Immunosorbent Assay
qPCR	Quantitative Polymerase Chain Reaction
RT-PCR	Reverse Transcription Polymerase Chain Reaction
RT Assay	Reverse Transcriptase Assay
PBS	phosphate-buffered saline
