# Supplementary material for: Real-Time PCR Assay to Quantify Moloney Murine Leukemia Virus in Mouse Cells
Source: Microorganisms. 2025 May 29;13(6):1268. doi: 10.3390/microorganisms13061268 (PMC12194895; doi:10.3390/microorganisms13061268)
Supplement: Supplementary file 1 [file microorganisms-13-01268-s001.zip › Supplementary Figure S1 - Re.pdf]

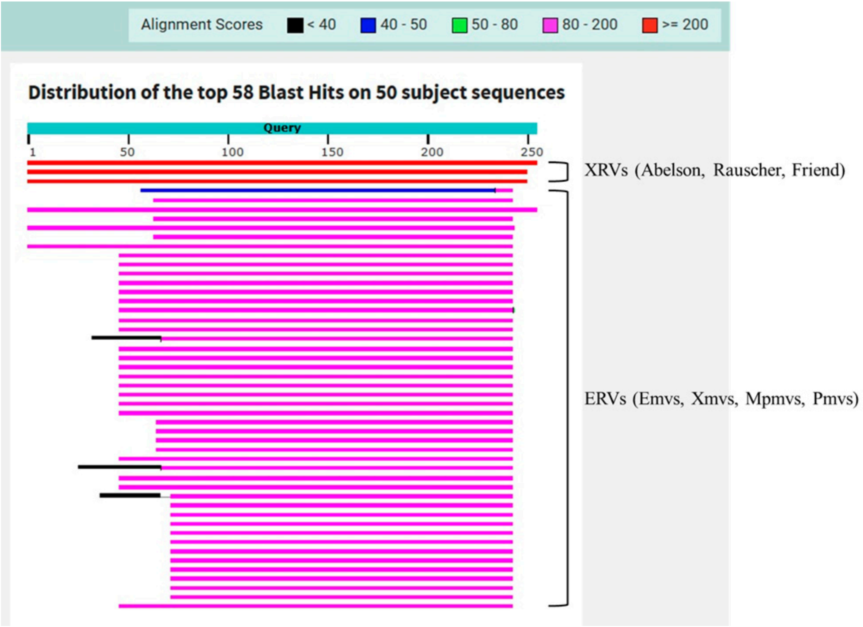

Supplementary Figure S1. Graphic summary of comparative sequence analysis between M-MuLV and other MuLVs. The nucleotide sequence from the psi/*gag* region of M-MuLV (RefSeq J02255.1, nt 550–802) was analyzed using the NCBI Blast 2 sequences program [26] to compare its similarity with 3 endogenous and 47 exogenous MuLV sequences. Accession numbers and query sequences for MuLVs are provided in Supplementary Material 2. The bars in the figure represent the regions used for alignment, and the alignment scores indicate the degree of sequence similarity between the M-MuLV reference and each MuLV subject sequence.
